# Effect of Echocardiographic Epicardial Adipose Tissue Thickness on Success Rates of Premature Ventricular Contraction Ablation

**DOI:** 10.4274/balkanmedj.galenos.2019.2019.4.88

**Published:** 2019-10-28

**Authors:** Selçuk Kanat, Bilge Duran Karaduman, Ahmet Tütüncü, Erhan Tenekecioğlu, Ferit Onur Mutluer, Nihal Akar Bayram

**Affiliations:** 1 Clinic of Cardiology, Bursa Yüksek İhtisas Training and Research Hospital, Bursa, Turkey; 2 Clinic of Cardiology, Ankara Atatürk Training and Research Hospital, Ankara, Turkey; 3 Department of Cardiology, Koç University Hospital, İstanbul, Turkey; 4 Department of Cardiology, Yıldırım Beyazıt University School of Medicine, Ankara, Turkey

**Keywords:** Ablation, epicardial adipose tissue, premature, ventricular complex

## Abstract

**Background:**

Idiopathic premature ventricular contractions are frequently detected ventricular arrhythmias, and radiofrequency ablation is an effectively treatment for improving symptoms and eliminating premature ventricular contractions. Studies have reported a relationship between an elevated epicardial adipose tissue thickness and myocardial structural pathologies. However, the association between epicardial adipose tissue thickness and success rates of premature ventricular contraction ablation has not yet been investigated.

**Aims:**

To assess the relationship between epicardial adipose tissue thickness and success rates of premature ventricular contraction ablation.

**Study Design:**

Retrospective case-control study.

**Methods:**

This study enrolled a total of 106 consecutive patients who have had a high premature ventricular contraction burden of >10,000/24-h assessed using ambulatory Holter monitorization and underwent catheter ablation. A frequency of premature ventricular contractions of more than 10,000/day was defined as frequent premature ventricular contraction. Epicardial adipose tissue thickness was measured using 2D transthoracic echocardiography. A successful ablation was defined as >80% decrease in pre-procedural premature ventricular contraction attacks with the same morphology during 24-h Holter monitorization after a 1-month follow-up visit from an ablation procedure.

**Results:**

Successful premature ventricular contraction ablation was achieved in 87 (82.1%) patients. Epicardial adipose tissue thickness was significantly higher in patients with unsuccessful ablation (p<0.001). Procedure time, total fluoroscopy time, and radiofrequency ablation time were statistically higher in the unsuccessful group (p<0.001). Stepwise multivariate logistic regression analysis showed that epicardial adipose tissue thickness and pseudo-delta wave time were independently associated with procedural success (both p values <0.001). In the receiver-operating curve analysis, epicardial adipose tissue thickness was found to be an important predictor for procedural success (area under the receiver-operating characteristic curve= 0.85, p=0.001), with a cutoff value of 7.7 mm, a sensitivity of 92%, and a specificity of 68%.

**Conclusion:**

Epicardial adipose tissue thickness is higher in patients with premature ventricular contraction ablation failure, which may be indicative of procedural success.

Idiopathic premature ventricular contractions (PVCs) are frequently observed ventricular arrhythmias. The assessment and treatment of PVCs are complex and highly dependent on the clinical scenario (1). The outflow tracts, both right outflow tracts and left outflow tracts and, are the best known origin of idiopathic PVCs. Idiopathic ventricular tachycardia or frequent PVCs from the right ventricular outflow tract generally occur in structural cardiac disease. Other sites for PVCs include the His-Purkinje system, the annuli of the aortic, pulmonary, and both atrioventricular valves, endocavitary structures, moderator band, and false tendons (2). Frequently occurring PVCs are generally symptomatic, and they have also been known to cause arrhythmia-induced cardiomyopathy. The frequency of PVCs per 24 h, which is related to decreased left ventricular function, has been described as attacks of >10%-25% of the sum of cardiac beats (3,4). Radiofrequency ablation is an effective treatment that has been used to improve symptoms and eliminate PVCs (5). The presence of remarkable symptoms with extremely frequent PVCs under medical therapy has been considered as an indication for ablation (6). Furthermore, the presence of PVC induced cardiomyopathy with or without symptoms is another indication for catheter ablation.

Epicardial adipose tissue (EAT) is a true visceral adipose deposit of the heart (7). EAT is not only adjacent to the atria, but it is also associated with autonomic ganglia and is the probable mechanism leading to arrhythmia (8). Several studies have demonstrated that EAT thickness is associated with some common disorders such as increased left ventricular mass, atherosclerosis, arterial stiffness, hypertension, and atrial fibrillation (9). Additional studies that have been conducted recently have emphasized the presence of a relationship between EAT thickness and atrial fibrillation existence and its postablation recurrence (10). Furthermore, one of the first reports in this context also demonstrated a significant association between increased EAT thickness and ventricular tachycardia recurrence associated with structural heart disease after catheter ablation (11). However, the association between EAT thickness and success rates of idiopathic PVC ablation remains unknown. Therefore, this study was conducted to evaluate the association between PVC ablation success rates and EAT thickness.

## MATERIALS AND METHODS

### Patient selection and study protocol

A total of 106 consecutive patients who underwent catheter ablation for idiopathic PVCs between January 2015 and July 2018 were enrolled in this study. Inclusion criteria were as follows: 1) patients with frequent PVCs as indicated by a total PVC count of >10,000 beats during 24-h Holter electrocardiography monitoring, 2) patients having symptoms or patients with symptomatic PVC-cardiomyopathy, 3) patients who were resistant to antiarrhythmic drugs, beta blockers, or nondihydropyridine calcium channel blockers (for at least 3 months), and 4) patients aged >18 years. The procedure of 24-h Holter recording was done again after 1 month of follow-up. The ablation procedure was considered to be successful when there was >80% decline in PVCs with the same morphology during 24-h Holter monitorization at the 1-month follow-up. Exclusion criteria of this study were follows: patients with coronary artery disease, including previous myocardial infarction, percutaneous coronary intervention, history of coronary artery bypass surgery, and structural cardiac disease, which were detected by exercise stress testing, coronary angiography, cardiac multidetector computed tomography, and/or radionuclide scans after proper indications, and patients with dilated or hypertrophic cardiomyopathy and valvular heart diseases (except mild form).

Demographic, clinical, and laboratory characteristics of the study patients were recorded from patient files. Electrocardiography or 24-h Holter recording electrocardiography data of the entire study population were also obtained. The study protocol was approved by the local institutional ethics committee.

### Echocardiographic examination

A transthoracic echocardiogram was performed using a commercially available equipment (Philips iE33 2006, USA) with 2D-guided M-mode features. The echocardiographic examination was conducted with the patient in the left lateral decubitus position. Parasternal long- and short-axis views and apical views were used as standard imaging windows. Baseline parameters such as both left ventricular end-diastolic and end-systolic volumes and left ventricular ejection fraction (calculated using modified Simpson’s method) were measured in the apical four-chamber view. All echocardiographic examinations were performed by a physician who was unaware of the study results.

**Measurement of EAT thickness:** Parasternal longitudinal and transverse parasternal views were used to measure the EAT thickness on the right ventricular free wall, and the mean value of both measurements was calculated. An echo-free space between the outer wall of the myocardium and the visceral layer of the pericardium was defined as an epicardial fat tissue. Its thickness was measured perpendicularly on the free wall of the right ventricle at end-systole in three cardiac cycles according to a predefined method (12). Data of the 106 patients were evaluated by two experienced cardiologists. Intra- and inter-observer variabilities for EAT thickness were 4.6% and 5.7%, respectively. Epicardial fat measurements from both long- and short-axis views were concordant.

### Electrophysiological study and radiofrequency catheter ablation procedure

All antiarrhythmic drugs, except amiodarone, were discontinued for five half-lives before the procedure. A decapolar mapping catheter (6F, 110 cm, Inquiry™, St. Jude Medical, St. Paul, Minnesota, USA) was inserted through the right femoral vein and placed in the coronary sinus under fluoroscopic guidance. A standard transvenous 6-F quadripolar catheter (6F, 110 cm, Marinr^®^ SC Series, Medtro nic, Minneapolis, MN, USA) was placed at the right ventricular apex. Endocardial signal and surface electrocardiography data were recorded using the EP Tracer device (Medtronic, Inc., USA). A 3D electroanatomic map was plotted using the CARTO 3 D Mapping System (Biosense-Webster, CA, USA), NAVX (St Jude Medical, MN, USA) or by noncontact mapping (Ensite Array, St Jude Medical). Ablation was performed using an irrigated-tip catheter contact sense catheter (Thermo-cool-SmartTouch, Biosense-Webster, Inc., CA, USA), an open-irrigated noncontact sense catheter (3.5-mm tip Thermocool or Thermocool SF, Biosense-Webster), and a FlexAbility catheter (Endosense/Abbott, St. Paul, Minnesota, USA). Activation mapping or pace mapping was used to identify potential sites for ablation. If no PVCs were detected or in case of only rare PVCs, intravenous isoproterenol was administered to facilitate the detection of PVCs. Acute procedural success was defined as no occurrence of spontaneous or inducible PVCs assessed using an isoproterenol infusion for a 30-min observation period after the final ablation lesion and the absence of the predominant PVC in the 24-h period postablation.

### Statistical analysis

One-sample Kolmogorov–Smirnov test was used for assessing whether the variables exhibited normal distribution. Variables were represented as mean ± standard deviation (minimum:maximum) or median (minimum:maximum) values. Based on the normality test results, an independent samples *t*-test or the Mann–Whitney U test was used for comparing the study groups. Categorical variables were compared by the chi-square test or Fisher’s exact test. Receiver-operating characteristic curve analysis was performed to estimate the sensitivity and specificity of EAT thickness levels for predicting the success rate of the procedure. Area under the receiver-operating characteristic curve values with 95% confidence intervals were reported. The relationship among continuous variables was examined using a correlation analysis, and Spearman’s correlation coefficient was computed. Logistic regression analysis was used to examine the associations among procedural success rates, EAT thickness, and other variables. Variables with a p value of <0.1 in the univariate logistic regression analysis were included in a multivariate logistic regression analysis. SPSS (IBM Corp. Released 2012. IBM SPSS Statistics for Windows, Version 21.0. Armonk, NY: IBM Corp.) and MedCalc for Windows (version 12.5, MedCalc Software, Ostend, Belgium) software were used for performing the statistical analysis, and the level of significance was set at p<0.05. Power analysis was done using a software program  (IBMM SPSS Sample Power, 2010, Chicago, USA) with power (1−β) set at 0.80 and α=05 (two-tailed).

## RESULTS

Table 1 shows the results of the comparison of baseline demographic characteristics, including past history of ablation, between patients with successful PVC ablation and those with unsuccessful ablation. Successful PVC ablation was achieved in 87 (82.1%) patients. In the remaining 19 (17.9%) patients, ablation was unsuccessful based on previously mentioned criteria. There was no difference in baseline characteristics between the study groups. The electrocardiographic and echocardiographic findings of the study participants are presented in Table 2. Left ventricular end-diastolic diameter, left ventricular end-systolic diameter, left ventricular end-diastolic diameter left ventricular end-diastolic volume, left ventricular end-systolic volume, and ejection fraction were not different between the two groups. The average duration of the maximum deflection index and the PVC maximum QRS duration were higher in the unsuccessful ablation group (p=0.045 and 0.034, respectively). EAT thickness was significantly elevated in the unsuccessful ablation group (p<0.001). Activation mapping revealed that the PVCs originated at the right ventricular outflow tracts in 43 patients (40.5%), aortic cusps in 37 patients (35%), papillary muscles in seven patients (6.6%), left ventricular summit in 12 patients (11.3%), multipl in three patients (1.9%), and other locations in three patients (2.8%) (Table 3). Procedure-related complications occurred in six patients as follows: cardiac tamponade in one patient each from both groups, one transient ischemic attack, and three hematomas in the successful ablation group. The effusion was self-limiting and managed conservatively in patients with successful ablation. In other patient, the pericardial effusion and the consequent tamponade were timely managed by pericardiocentesis. The suspected site of localization included only an epicardial summit region in this patient. Transient ischemic attack, which becomes evident as a visual impairment immediately after the procedure in a patient, was managed conservatively, and the clinical picture was self-limiting with complete recovery. The suspected site of localization was a left ventricular papillary muscle in this patient. The procedure duration time, the total fluoroscopy time, and the net duration of radiofrequency time were statistically higher in the unsuccessful group as expected (p<0.001).

Epicardial fat thickness and fluoroscopy duration showed a significantly positive correlation between each other for all cases (rs=0.31; p=0.008) (Table 4). In the stepwise multivariate logistic regression analysis, EAT thickness and pseudo-delta wave time were found to be independently associated with procedural success rates (both p values <0.001) (Table 5).

**Figure 1:**
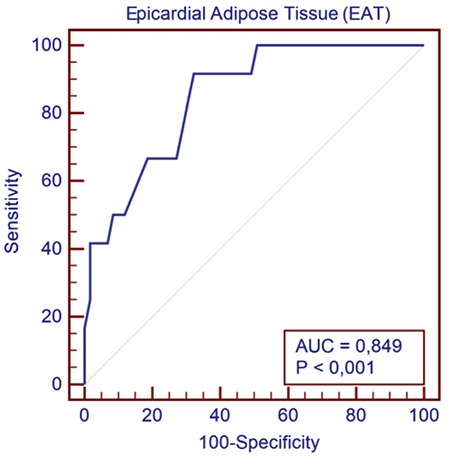
Receiver-operating characteristic curve for the prediction of procedural success using epicardial adipose tissue thickness. The area under the curve for epicardial adipose tissue thickness is 0.85 (95% CI: 0.74-0.92) with p<0.001. AUC: area under the curve; CI: confidence interval

In the receiver-operating characteristic analysis, the EAT thickness achieved an area under the receiver-operating characteristic curve of 0.85 (p=0.001) for predicting the procedural success (Table 6). A cutoff value for EAT thickness of 7.7 mm was considered for predicting procedural success rates, with a sensitivity of 92% and a specificity of 68% (Table 5, Figure 1).

## DISCUSSION

This study demonstrated that EAT thickness and fluoroscopy duration were significantly higher in the unsuccessful ablation group. In total, 42% of the PVCs were from the right outflow tracts, with 32% from the aortic cusps. Catheter ablation proved to be highly effective in eliminating these PVCs. Using a similar patient group as that of our study, another study reported that the 3-month success rate of ablation involving all idiopathic PVCs was 80% (13). This finding is similar to the success rate observed in our study. Finally, this study also demonstrated that EAT thickness was a strong predictor of procedural success in patients undergoing PVC ablation.

The success rate of ablation was found to be lower in patients with PVCs originating from the left ventricular summit region [6 (31.58%) versus 6 (6.70%) p=0.024]. Electrocardiographic and electrophysiological features were taken into consideration for determining whether the premature ventricular complexes were summit-folded (14,15). Cheng et al. demonstrated that for left ventricular summit ventricular arrhythmias, the prolonged pseudo-delta wave time (PdW>53 msn), the intrinsicoid deflection time (IDT>74 msn), and the maximum deflection index (MDI>0.45) indicated an epicardial origin. PVCs originating from this region can be eliminated by applying radiofrequency catheter ablation within the great cardiac vein or using a subxiphoid transpericardial access. Similar to the results of our study, Yamada et al. (16) demonstrated that radiofrequency catheter ablation success rate is lower because left ventricular summit is close to coronary arteries and/or epicardial adipose tissue.

PVCs are common causes of palpitations. The detection of rare PVCs during 24-h ambulatory Holter monitoring is highly frequent and generally accepted as a common finding. The primary indications for catheter ablation of PVCs are being symptomatic under medical therapy and having an left ventricular dysfunction (6). Radiofrequency catheter ablation of frequent idiopathic PVCs has been accepted as a convenient, beneficial, and effective treatment method for symptomatic frequent PVCs and suspected PVC induced cardiomyopathy. Zhong et al. (17) demonstrated in their recent study that a mean decrease in PVC burden was significantly higher in the ablation group than in the medical therapy group (-21,779 per 24 h mean PVC decrease in the ablation group compared to -8376 per 24 h in the medical therapy group, p<0.001).

It has been well established that EAT can be considered as a form of visceral adipose tissue (17). In addition to systemic effects, EAT exerts paracrine effects. It releases proinflammatory cytokines such as tumor necrosis factor-alpha, interleukin-1-beta, and interleukin-6, which can cause variable irreversible changes in the myocardium structure, including myocardial fibrosis (18). The association between EAT thickness and atherosclerosis, hypertension, impaired coronary flow reserve, arterial stiffness, and atrial fibrillation has been reported in some studies (19,20,21,22,23). Wong et al. (24) examined epicardial fat using cardiac magnetic resonance in 130 patients and demonstrated that arterial pericardial fat volumes are associated with the prevalence and severity of atrial fibrillation and the recurrence after radiofrequency ablation. It is also known that EAT thickness is associated with higher levels of inflammatory cytokines, and a higher inflammatory state can subsequently lead to a higher structural and electrical remodeling of the heart and finally a higher incidence of serious ventricular arrhythmias and sudden cardiac death (18). Wu et al. (25) demonstrated an association between pericardial adipose tissue and the development of ventricular fibrillation/ventricular tachycardia in 50 patients with systolic HF. Recently, Shamloo et al. (11) also reported that magnetic resonance-measured epicardial fat volumes (right and left atrioventricular grooves, as well as anterior interventricular groove) were significantly higher in the ventricular tachycardia recurrence group than in the non-recurrence group. However, unlike our study, sustained monomorphic ventricular tachycardia in patients with structural heart disease was discussed in that study.

Several mechanisms may be responsible for establishing an association between PVC ablation success rate and EAT thickness. The mechanism of PVCs may be structural and ultrastructural changes, which are caused by EAT. Myocardial structural impairment such as advanced remodeling and the resulting fibrosis may lead to alterations in action potential characteristics, which trigger the heart to develop PVCs (26). Kırış et al. (27) reported an elevated EAT thickness in the setting of PVCs. They also found an independent association between EAT thickness and frequent PVCs. Akyüz et al. (28) found an elevated epicardial fat thickness in patients with fragmented QRS compared to that in controls, which was considered as an indicator for myocardial fibrosis or scarring in a variety of diseases. Voulgari et al. (29) reported the presence of a relationship between left ventricular arrhythmogenicity and low levels of inflammation in metabolic syndrome. The association between EAT thickness and ablation success may also depend on other possible mechanisms, such as the effects of adipose tissue on impedance and even the role of the autonomic nervous system in EAT. EAT, the visceral fat repository in the heart, contains intrinsic cholinergic and adrenergic nerves that interact with the extrinsic cardiac parasympathetic and sympathetic nervous systems. These EAT nerves represent a significant source of norepinephrine and epinephrine. White (30) reported that an increase in EAT volume correlates with a hyperactive adrenergic signaling resulting from an increased catecholamine biosynthesis. EAT thickness is also associated with higher levels of inflammatory cytokines, and a higher inflammatory state can subsequently lead to a higher structural and electrical remodeling of the heart and finally a higher incidence of arrhythmia. Consequently, an increased adrenergic activity may augment the frequency of PVCs that may lead to increased resistance to ablation therapy.

### Study limitations

The present study had some limitations. First, we had a small study population, which was derived from a single center. Second, advanced imaging techniques such as magnetic resonance imaging and computed tomography were not used to detect EAT. Although two-dimensional echocardiography is a useful imaging modality for evaluating EAT thickness, which significantly correlates with magnetic resonance imaging and computed tomography, these three techniques are not useful in routine clinical practice due to their lack of cost-effectiveness, the time-consuming feature, and the radioactive effect, respectively. Third, we did not evaluate the correlation between EAT thickness and inflammatory markers or cytokines in our study. Finally, the specificity of EAT for predicting the success rate of the ablation procedure, which was detected by the receiver-operating characteristic curve analysis, was low. Results of an ablation procedure may depend on several factors, which are related to certain inflammatory markers or structural changes of the heart such as EAT thickness. However, a limited number of patients may also decrease the specificity of EAT in some disease settings. Moreover, there are also other factors that could affect the procedural success and the sensitivity and specificity of EAT.

To our knowledge, our study is the first to demonstrate a useful effect of transthoracic echocardiography-derived EAT thickness in identifying the success rate of PVC ablation. EAT thickness was found to be increased in the unsuccessful ablation group. Furthermore, EAT thickness was found to be a better and independent predictor of procedural success in PVC ablation. The causal relationship between EAT thickness and pathogenesis of PVCs requires further studies with a larger population.

## Figures and Tables

**Table 1:**
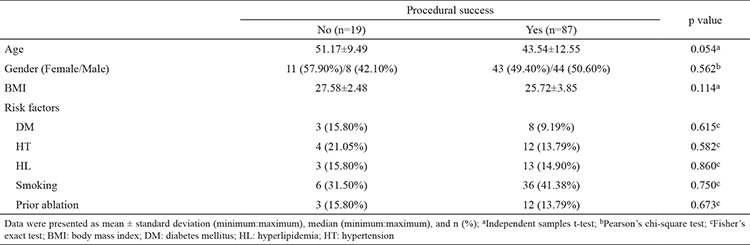
Baseline characteristics of patients

**Table 2:**
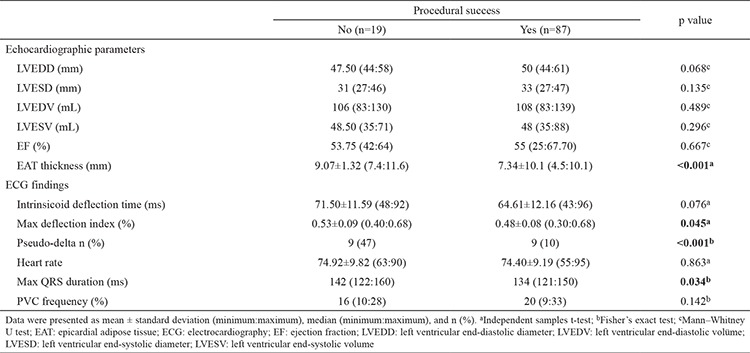
Echocardiographic parameters and electrocardiography findings according to procedure success

**Table 3:**
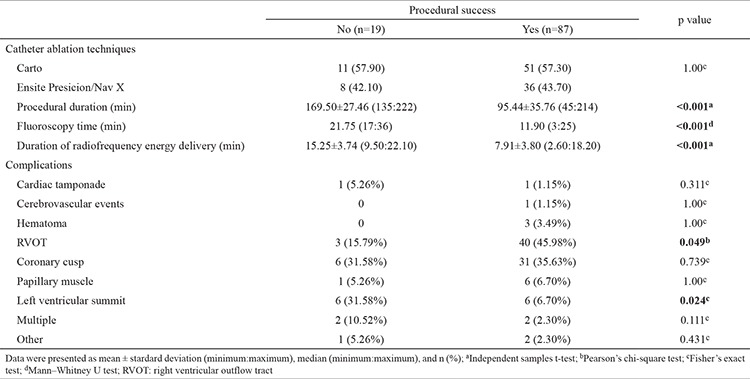
Details and complications of ablation procedures

**Table 4:**
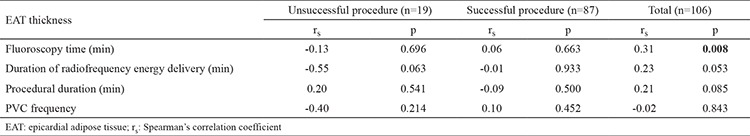
Correlation between epicardial adipose tissue thickness and procedural details

**Table 5:**
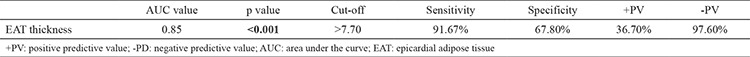
Receiver-operating characteristic curve for the prediction of procedural success using epicardial adipose tissue thickness

**Table 6:**
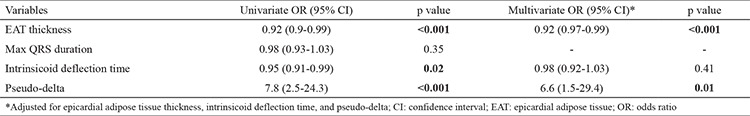
Association between procedural success and multiple variables in univariate and multivariate logistic regression analyses
